# Prospective changes in anemia are associated with the incidence and persistence of sarcopenia among older Mexican adults

**DOI:** 10.3389/fnut.2024.1323450

**Published:** 2024-03-13

**Authors:** Vanessa De La Cruz-Góngora, Aaron Salinas-Rodriguez, Betty Manrique-Espinoza

**Affiliations:** Center for Evaluation and Surveys Research, National Institute of Public Health, Cuernavaca, Mexico

**Keywords:** anemia, sarcopenia, incidence, prevalence, older adults

## Abstract

**Background:**

Low hemoglobin levels are a significant biomarker in the prognosis of sarcopenia. Anemia and sarcopenia are frequent and disabling conditions in the older adult population, but little is known about the role of anemia in the onset and progression of sarcopenia. This study aimed to determine whether prospective changes in anemia are associated with the incidence and persistence of sarcopenia.

**Methods:**

Data come from the second and third waves (2014, 2017) of the World Health Organization (WHO) Study on global AGEing and adult health (SAGE) in Mexico. SAGE-Mexico is a dynamic cohort with national representativeness, including a follow-up sample and new enrollments. For this study, 1,500 older adults (aged 50 or above) with measurements in both waves were included. Sarcopenia was defined as having low muscle quantity and either/both slow gait speed and weak handgrip strength. Anemia was defined according to hemoglobin concentrations, adjusted for altitude, as recommended by the WHO, <120 g/L for women and <130 g/L for men. Multinomial logistic regression was used to estimate the association between anemia and prospective changes in sarcopenia.

**Results:**

The baseline prevalence of anemia was 17.4%, and that of sarcopenia was 12.1%. The incidence and persistence of anemia were 10.6% (95% CI: 7.3–15.0%) and 6.9% (95% CI: 4.7–9.8%), respectively, and for sarcopenia, they were 5.3% (95% CI: 3.7–7.7%) and 9.2% (95% CI: 6.4–13.0%), respectively. Incident anemia was associated with incident (RRR = 3.64, 95% CI: 1.18–11.19) but not with persistent (RRR = 0.75, 95% CI: 0.18–3.20) sarcopenia. Persistent anemia was significantly associated with persistent (RRR = 3.59, 95% CI: 1.14–11.27) but not incident (RRR = 1.17, 95% CI: 0.30–4.54) sarcopenia.

**Conclusion:**

Changes in anemia are significantly associated with incident and persistent sarcopenia. Primary actions to promote a healthy diet rich in antioxidants, high-quality proteins, and micronutrients, as well as moderate physical activity and maintaining a healthy weight, are crucial for the aging population to delay the deleterious effects of anemia and sarcopenia.

## 1 Introduction

The decline in physical function in aging is one of the main drivers of the onset of sarcopenia, a geriatric syndrome characterized by poor physical performance, low strength, and low mass muscle (1), with function loss accelerating three times faster than muscle loss and increasing the appearance of disability falls and frailty (2). Among older adults, sarcopenia is a frequent condition, with prevalence rates ranging from 0–15% in healthy older adults and 2–34% in geriatric outpatients (3). For Mexico, the prevalence ranges from 9.3 to 33.6% (4). Empirical evidence has shown that sarcopenia negatively affects older people’s cognitive function, quality of life, and survival rates (5–7). Anemia is also a frequent condition in older adults. The global prevalence of anemia for this age group has been estimated at 23.9 and 28.8% for Mexico (8, 9). Like sarcopenia, anemia has been identified as a risk factor for critical health indicators such as poor physical performance, quality of life, disability, and mortality (10).

Previous studies have analyzed the association between anemia and sarcopenia with inconclusive results. A recent meta-analysis with 98,502 community-dwelling participants aged 60+ years that aimed to identify the factors associated with sarcopenia reported that anemia was significantly associated with sarcopenia. However, for this last association, only two Japanese studies with 2,408 older adults were included (11). Meanwhile, studies on the specific association between anemia and sarcopenia have mixed results. One study with Taiwanese older adults reported a significant association (12), but for another two studies (Japan and Taiwan), the association was no longer significant (13, 14). Two additional prospective cohort studies with older Australian men and older American adults, have shown that anemia increased the risk in the decline of physical performance and sarcopenia (15, 16).

Despite this incipient prior evidence, knowledge gaps still must be addressed. This is mainly because the results have been generated either by cross-sectional or longitudinal studies that have yet to explore the influence that changes in anemia have on changes in sarcopenia prospectively. Moreover, whether anemia has a role in the onset or persistence of sarcopenia has yet to be explored, and the evidence from the Latin American population is null, even though structural risk factors might be higher than those in high-income countries (HIC). Particularly, anemia rates in populations from low- and middle-income countries are higher and also have different causes compared to those in HIC (17). In fact, it remains uncertain whether the accumulated risk of persistent anemia affects sarcopenia over time.

Anemia and sarcopenia are frequent conditions in the older adult population, and both are modifiable risk factors. In contexts where the double burden of malnutrition exists (particularly in low- and middle- income countries such as Mexico), early identification of biomarkers as a prognosis of the onset of sarcopenia is crucial to promote early actions and delay their pervasive consequences. This study aimed to evaluate whether prospective changes in anemia were associated with an increasing risk of sarcopenia over a 3 years follow-up in a representative national sample of older Mexican adults.

## 2 Materials and methods

### 2.1 Population and sample

**Figure 1:**
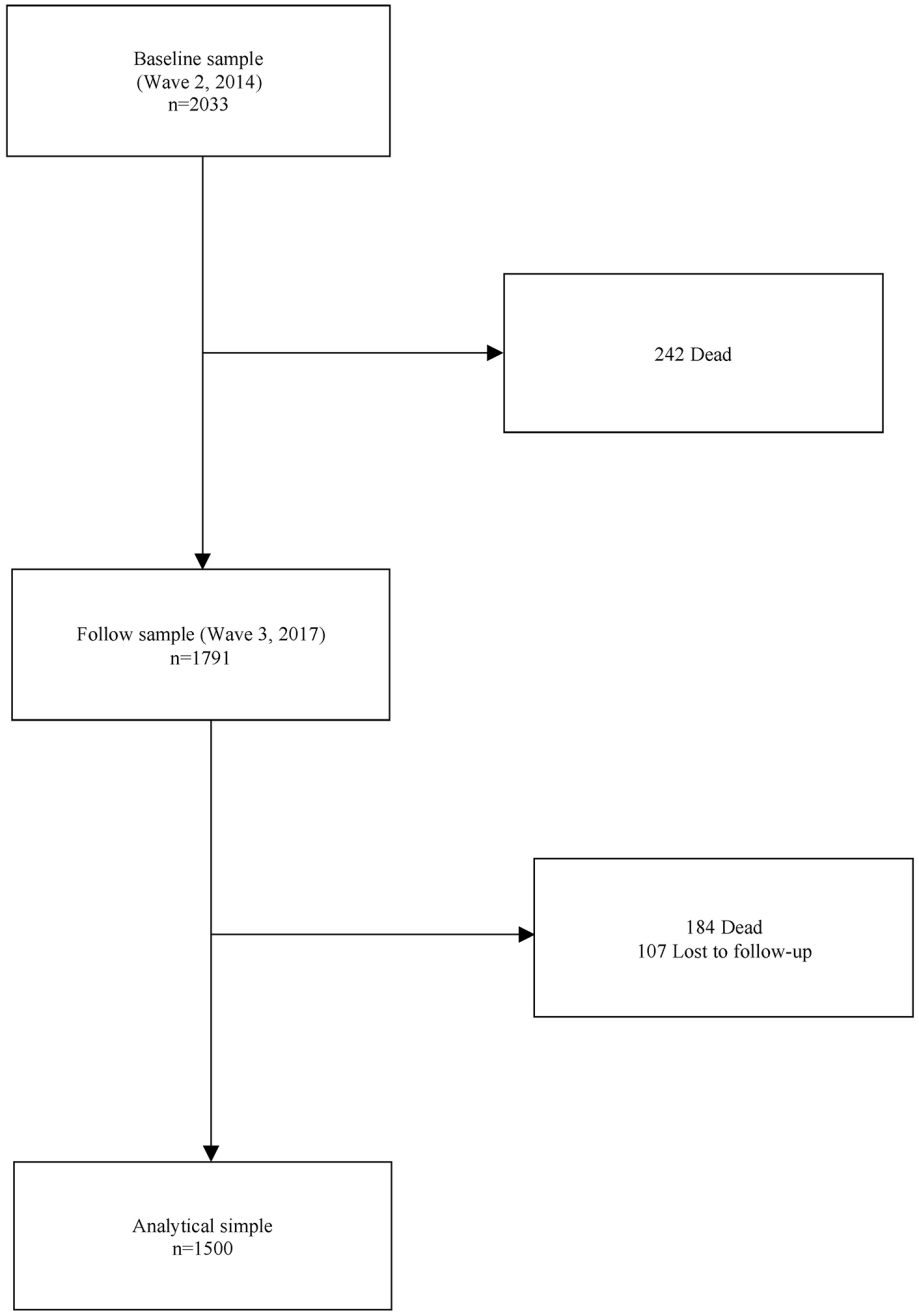
Study population and analytical sample.

We used data from the World Health Organization (WHO) Study on global AGEing and adult health (SAGE) in Mexico. A multicountry, longitudinal study, SAGE, was based on nationally representative samples of individuals aged 50+ years in six countries: China, Ghana, India, Mexico, Russia, and South Africa. Details of the study design have been published elsewhere (18). The SAGE-Mexico study and sample (cross-sectional and longitudinal) have been previously described (19, 20). Briefly, SAGE-Mexico included a sample of follow-up respondents from SAGE Wave 0, a baseline cohort created during the 2002–2004 World Health Survey, and new respondents. Proxy respondents were identified for respondents who were unable to provide reliable responses or due to poor health. Also, SAGE-Mexico is a nationally-representative sample of older Mexican adults (50+ years) collected using a stratified multistage cluster sample design. In-person interviews were used to collect household and individual level data for each wave. Specifically, two strata were defined by dwelling area (urban and rural). Within these strata, the Basic Geo-Statistical Areas (AGEB by its Spanish acronym) defined by the Mexican National Institute of Statistics were used as primary sampling units (PSU). Households within PSU were randomly selected and constituted the secondary sampling units. Household weights were post-stratified by AGEB according to population census projections. Finally, individuals within households made up tertiary sampling units. Individual weights were post-stratified by sex and age-groups (18–34, 35–49, 50–59, 60–105) according to the census projections. Sample size and date for each wave were: Wave 1 (baseline data) was collected in 2009 with 2,306 respondents. Wave 2 was carried out in 2014 with 2,033 interviews, and Wave 3 was carried out in 2017 with 1,791 participants (plus 618 newly enrolled individuals). For this study, we use data from the most recent waves of SAGE-Mexico (2014 and 2017). The analytical sample consisted of 1,500 older adults who had measurements of anemia and sarcopenia in both waves, with an overall response rate of 84% (Figure 1). Baseline differences between the final sample and excluded participants were observed. Older adults without follow-up measurements were older, had a lower prevalence of sarcopenia and smoking, were mostly women and were mainly from rural areas (*p* < 0.05).

### 2.2 Outcome

#### 2.2.1 Sarcopenia

The presence of sarcopenia was defined, according to previous studies using the Mexico-SAGE data, as having low skeletal muscle mass (SMM), reflected by lower skeletal muscle mass index (SMI), and either or both slow gait speed and weak handgrip strength. We recently provided a detailed description of this measurement (21). We observed four groups according to changes in sarcopenia: no sarcopenia in both measurements (none), sarcopenia at baseline and not in follow-up (recovery), sarcopenia only in follow-up (incident), and sarcopenia in both measures (persistent). Since the proportion of individuals in the recovery category was low (≈2%), the none and recovery categories were joined, leaving the following categories for sarcopenia: none/recovery, incident, and persistent.

### 2.3 Main exposure

#### 2.3.1 Anemia

Dried blood spot (DBS) samples were extracted with a finger lancet and collected on standard Whatman 903 filter paper. The samples were analyzed after 24 h of drying at room temperature. A 6 mm spot was punctured from the filter paper and eluted for 14 h in 400 μL of MULTIAGEN Hemoglobin Denaturant. Hemoglobin (Hb) was run via blood using the Abbott Architect CI8200 chemistry analyzer. The total Hb was determined by measuring absorbance at 604 nm. Anemia was defined according to Hb concentrations, as recommended by the WHO, <120 g/L for women and <130 g/L for men. Four groups were also defined in relation to observed changes in anemia: none, recovery, incident and persistent.

### 2.4 Covariates

The following health, socioeconomic and lifestyle baseline variables were used as potential confounders: sex (female = 1), age, number of years of formal education, and dwelling area (rural = 1). The socioeconomic status (SES) of the household was derived using the WHO standard approach to estimate permanent income from household ownership of durable goods, dwelling characteristics (type of floors, walls, and cooking stove), and access to services such as water, sanitation, and electricity (22). SES was included as a continuous variable, with higher values indicating better SES. Multimorbidity was included as a dichotomous variable defined as the presence of two or more chronic noncommunicable conditions from the list of 12 chronic diseases included in the SAGE study. The operational definitions of these diseases have been published elsewhere (23). The body mass index (BMI) was calculated using weight (kg) and height (cm) [BMI = Weight (kg)/Height (m^2^)] and was incorporated into the analysis as a continuous variable. The C-reactive protein (mg/L) DBS values were included as a potential inflammation marker. Physical activity was assessed with the Global Physical Activity Questionnaire (GPAQ), which classifies older adults into three categories (low, moderate, and high physical activity) based on reported time spent in moderate or vigorous activities during work, recreational/leisure time, and transportation. As for tobacco use and alcohol consumption, respondents were asked if they had ever used tobacco or consumed alcohol, and if participants answered affirmatively the frequency of use was recorded (24). With this information tobacco use was categorized as *never*; *ever smoked*, *no longer*; *current smoker*, *not daily*; *current smoker*, *daily*; and alcohol consumption as *never*; *ever drinker*, *no longer*; current drinker, low risk; *current drinker*, *high risk*. Fruit and vegetable consumption (servings per day), and sedentary behavior (daily sitting hours) were self-reported. Food insecurity (FI) was operationalized using items adapted from similar items in food security questionnaires of the US Household Food Security Survey Module and National Health and Nutrition Examination Survey (NHANES) Food Security module. In line with previous SAGE studies, FI was coded as severely food insecure, moderately food insecure, and food secure (25).

### 2.5 Statistical analyses

Baseline characteristics are presented in percentages and means (standard deviation) as appropriate. Health and sociodemographic characteristics related to sarcopenia groups were compared using chi-square or ANOVA tests. We used a multinomial logistic regression model to estimate the association between changes in anemia and sarcopenia. Relative risk ratios (RRRs) and 95% confidence intervals were reported. Associations were considered significant if *p* < 0.05. All statistical analyses considered the sampling weights and were performed using STATA version 18.0 software (StataCorp. 2023. Stata Statistical Software: Release 18. College Station, TX: StataCorp LLC.). This study was conducted following the STROBE guidelines for reporting cohort studies (STROBE checklist is reported in Supplementary material).

### 2.6 Ethics

This investigation was conducted in accordance with the ethical standards laid down in the 1964 Declaration of Helsinki and its later amendments (as revised in 1983). The study was approved by the research and ethics committees of the National Institute of Public Health, Cuernavaca, Mexico (CI/2020/550). All subjects gave written informed consent.

## 3 Results

The baseline study sample included 1,500 older adults, with a mean age of 61.2 years (SE = 0.49). A total of 54.5% were female, with a mean of 6.2 years of formal education (SE = 0.38), 13.8% had severe food insecurity, and 61.7% had multimorbidity. For lifestyle variables, 45.8% performed a low level of physical activity and had a mean daily sitting hours of 2.7 (SE = 0.14). Finally, 37.5% had never smoked, and 71.4% had never consumed alcohol (Table 1).

**Table 1:** Baseline characteristics by sarcopenia groups.

	Sarcopenia^a^				*p*-value^b^
	Total *n* = 1,500	None/recovery *n* = 1,238	Incident *n* = 99	Persistent *n* = 163	
**Exposure**					
Anemia (%)	17.39	15.60	30.51	26.41	0.07
**Covariates**					
Sex (female, %)	54.48	55.75	44.70	49.47	0.44
Age (years)	61.19 (0.49)	59.77 (0.45)	69.02 (1.55)	69.86 (1.87)	<0.01
Body mass index [weight (kg) ÷ squared height (meters)]	28.69 (0.25)	28.53 (0.27)	29.51 (1.03)	29.72 (0.71)	0.27
Multimorbidity (2 or more chronic conditions) (%)	61.66	59.97	61.68	77.38	0.09
C-reactive protein (mg/L)	1.42 (0.09)	1.42 (0.10)	1.67 (0.46)	1.22 (0.22)	0.61
**Physical activity (%)**					
Low	45.81	44.41	67.63	46.20	
Moderate	25.85	26.27	21.84	24.24	
High	28.34	29.32	10.53	29.56	0.19
Sedentary behavior (daily sitting hours)	2.65 (0.14)	2.67 (0.15)	2.19 (0.41)	2.78 (0.33)	0.53
**Alcohol consumption (%)**					
Never	37.09	35.52	50.02	44.19	
Ever drinker, no longer	47.27	48.52	42.67	38.37	
Current drinker (low risk)	10.33	10.75	1.66	11.46	
Current drinker (high risk)	5.30	5.21	5.66	5.98	0.57
**Tobacco use (%)**					
Never	71.41	71.80	67.80	69.81	
Ever smoked, no longer	13.51	12.99	12.08	19.15	
Current smoker, not daily	2.30	2.35	4.11	0.80	
Current smoker, daily	12.79	12.86	16.01	10.24	0.82
Fruits and vegetable consumption (number of portions per day)	2.82 (0.12)	2.81 (0.13)	3.26 (0.20)	2.69 (0.30)	0.10
**Food insecurity (%)**					
None	78.08	78.38	86.01	70.72	
Moderate	8.08	9.02	3.66	1.92	
Severe	13.84	12.60	10.33	27.37	<0.01
Schooling (years of formal education)	6.20 (0.38)	6.51 (0.42)	4.65 (0.61)	4.26 (0.64)	<0.01
Socioeconomic status (assets index)	0.13 (0.09)	0.19 (0.10)	0.16 (0.16)	0.21 (0.25)	0.03
Dwelling area (rural, %)	22.32	24.01	18.24	9.04	0.10

a Cells are percentages or means (std. error).

b *p*-value for chi-square or ANOVA tests.

The proportions of sarcopenia and anemia for each transition group are shown in Table 2. The cumulative incidence of sarcopenia was 5.3% (3.7–7.7%), and persistence was 9.2% (6.4–13.0%). Regarding anemia, the recovery rate was 12.0% (8.6–16.4%), the cumulative incidence was 10.6% (7.3–15.0%), and the persistence was 6.9% (4.7–9.8%).

**Table 2:** Proportion of older adults in each sarcopenia and anemia group.

		Estimator	CI 95%	
Sarcopenia	None/recovery	85.5	81.3	88.8
	Incident	5.3	3.7	7.7
	Persistent	9.2	6.4	13.0
Anemia	None	70.7	64.7	76.0
	Recovery	12.0	8.6	16.4
	Incident	10.6	7.3	15.0
	Persistent	6.9	4.7	9.8

Table 1 shows the exposure and covariate distribution by sarcopenia groups. Older adults with incident or persistent sarcopenia were older (*p* < 0.01), with a higher prevalence of food insecurity (*p* < 0.01), fewer years of schooling (*p* < 0.01), and poorer (*p* = 0.03) than individuals in the group with no/recovery sarcopenia. No significant differences were observed in health and lifestyle variables (Table 1).

Table 3 depicts the results of the multinomial logistic regression model. The cumulative incidence of anemia was significantly associated with incident sarcopenia (RRR = 3.66; 95% CI 1.18–11.19; *p* = 0.02) and persistent anemia with persistent sarcopenia (RRR = 3.59; 95% CI 1.14–11.27; *p* = 0.03). No significant associations were observed for recovery from anemia. Incident anemia was not significantly related to persistent sarcopenia, nor was persistent anemia related to incident sarcopenia.

**Table 3:** Results of the multinomial logistic regression models.

	Incident sarcopenia				Persistent sarcopenia			
	RRR	CI 95%		*p*-value^a^	RRR	CI 95%		*p*-value^a^
**Anemia (ref: none)**								
Recovery	0.54	0.20	1.43	0.22	1.05	0.26	4.26	0.95
Incident	3.66	1.18	11.19	0.02	0.63	0.20	2.06	0.45
Persistent	0.75	0.18	3.20	0.70	3.59	1.14	11.27	0.03

a Adjusted for covariates shown in Table 1.

**Figure 2:**
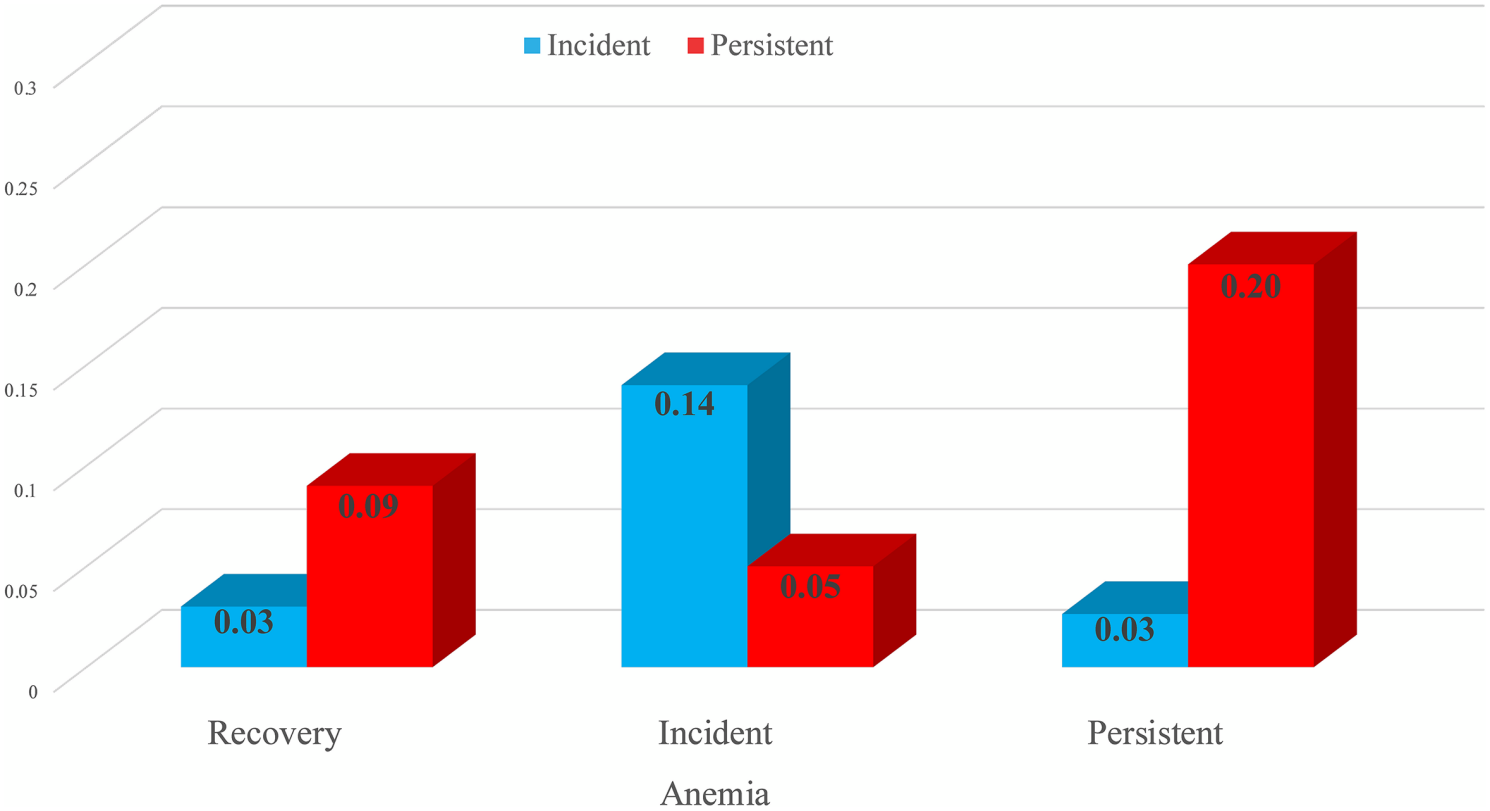
Conditional probabilities of incident and persistent sarcopenia.

Figure 2 shows the conditional probabilities of incident and persistent sarcopenia, given the observed transitions in anemia. Compared with the incident anemia group, older adults with persistent anemia were four times more likely to have persistent sarcopenia. At the same time, older adults with incident anemia had a 4.7 times greater chance of incident sarcopenia than individuals with persistent anemia.

## 4 Discussion

The results of this study provide evidence of the prospective changes in anemia and their association with incident and persistent sarcopenia in a representative sample of older adults in Mexico with longitudinal data that encompasses a 3 years follow-up. Incident anemia was consistently associated with a 4.7 times risk of incident sarcopenia, and persistent anemia had a four times greater probability for persistent sarcopenia compared to the absence of anemia in both measurements, baseline and follow-up.

A recent systematic review and meta-analysis reported that anemia was associated with a higher probability of sarcopenia (OR: 1.4, CI 95%: 1.06–1.82), although these results were obtained from only two studies (11). Regarding the incidence or persistence of sarcopenia, few cohort studies have explored their association with anemia, although placing greater emphasis on continuous hemoglobin levels (Hb). A cohort study with older Chinese adults found that an increase in Hb of 1 g/dL was associated with a lower rate (8%) of sarcopenia incidence over a 4 years follow-up (26). Another study with older Australian men also reported that a 1 g/dL increase in Hb was significantly associated with a reduction in the odds of sarcopenia throughout 5 years of study (OR = 0.71, CI 95% 0.61, 0.82). In the same study, moderate anemia (OR = 3.0, *p* < 0.01) had a stronger association than mild anemia (OR = 1.73, *p* < 0.01) (15). Another study with older American adults (71 years or older) showed that anemia was associated with a higher decline in physical performance over a 4 years follow-up (16). It is important to mention that this study did not specifically analyze sarcopenia but rather a series of physical performance tests (standing balance, a timed 2.4 m walk, and a timed test of five chair rises).

Our results are consistent with those reported in these studies since anemia was significantly associated with incident and persistent sarcopenia. However, our results are not entirely comparable since we explicitly explored whether prospective changes in anemia (incidence and persistence) influenced the incidence and persistence of sarcopenia. In this sense, our study provides new, robust, and specific information about the role of the incidence and persistence of anemia concerning incident and persistent sarcopenia. Accordingly, the incidence of anemia is a significant prognostic factor for the incidence of sarcopenia, while persistent anemia accounts for a high attributable proportion of persistent sarcopenia. However, the evidence on this relationship remains controversial, and future studies with controlled designs should confirm or refute these results.

Although the specific mechanisms underlying the association between anemia and sarcopenia are not well understood, it has been proposed that low hemoglobin levels reduce oxygen delivery to all cells, impairing skeletal muscle mitochondrial respiration. Mitochondria are the primary site of ATP production and lean muscle fuel metabolism (27). Myoglobin, the protein that carries oxygen to muscle tissue, is affected by anemia, generating local hypoxia in skeletal muscle. Consequently, anemia increases the risk of muscle fatigue, and chronic fatigue impairs muscle mobilization, leading to atrophy, affecting functionality, and increasing the risk of falls and disability (28). The observed association between anemia and sarcopenia in our study might also be partially explained by chronic low-grade inflammation since this has been identified as the main contributor to anemia in one study with older adults from the southern region of Mexico (29). Chronic inflammation also contributes to the loss of muscle mass, strength, and functionality (30), and it is a common shared pathway with anemia due to chronic disease and other geriatric syndromes (31).

Anemia and sarcopenia might also share a nutritional cause as etiology, given that both conditions have been linked through malnutrition (11, 32). Previous evidence has indicated that nutritional anemia could account for one-third of all anemias in older people. Aside from iron deficiency, nutritional anemia is associated with vitamin B12 (cobalamin), which is frequently related to dietary cobalamin malabsorption and vitamin B9 (folate) deficiency (33–35). The inadequate intake of nutrients, associated with the loss of appetite during aging, is a nutritional factor leading to poor protein intake, which can also cause iron deficiency and other nutritional anemias. As for older Mexican adults, the adequacy intake of some essential micronutrients, such as iron and zinc, is low (36). However, tortillas and beans are part of the traditional Mexican diet and are consumed in large amounts. They are sources of protein, micronutrients (nonheme iron), and inhibitors of iron absorption as phytates and tannins. Despite this, previous studies have shown that although the Mexican diet has a high iron content, its bioavailability is low, which, in turn, can affect iron status (37). Poor protein quality mainly reflects the low bioavailability of iron, B12, and other critical micronutrients involved in erythropoiesis and protein synthesis needed for the anabolism of muscle fibers. However, micronutrient deficiency appears to have a low contribution to anemia in older Mexican adults (29), and its impact on the risk of sarcopenia in this population is also unknown.

The study results should be interpreted in the presence of some limitations. Hb measurement was based on a capillary dried blood sample, which might misclassify anemia diagnosis, affecting those around the cutoff value. Nonetheless, Hb data were calibrated with venous blood to minimize bias due to measurement error (38). The SMI was estimated through a formula instead of more precise methods such as DXA or BIA; this could result in low sensitivity of subjects categorized with low mass muscle. In addition, we do not have any information regarding diet and protein quality, the etiology of anemia, or the treatment for anemia, which could help explain the risk in the incidence and persistence of sarcopenia. Reverse causality could also explain the results obtained. Older adults with persistent sarcopenia have specific characteristics, such as a higher prevalence of chronic comorbidities, that increase the risk of persistent anemia, with low-grade inflammation as a shared common pathway where anemia by chronic disease may arise.

## 5 Conclusion

The results of this study have important implications for research, clinical practice, and public health policies targeting older adults. Regarding health policies, addressing the primary needs of all people to reach food security is crucial. Most anemia cases in older Mexican adults have an inflammatory component secondary to chronic disease (29); therefore, secondary prevention should be reinforced to maintain autonomy in older adults. Primary actions to promote a healthy diet rich in antioxidants, high-quality proteins, and micronutrients, as well as moderate physical activity and maintaining a healthy weight, are vital for the aging population to delay the deleterious effects of anemia and sarcopenia. For clinical practice, according to clinical guidelines in Mexico, it is recommended to assess and determine the cause of anemia and treat it whenever feasible. Individuals with micronutrient deficiencies, such as iron, B12, and folate, should be given supplements to address the deficiency. For research, further studies should explore the effect of treating anemia on the onset of broad functional outcomes in older adults. In conclusion, the current study provides evidence that anemia is an independent risk factor for incident and persistent sarcopenia. Given that both conditions are highly prevalent and modifiable, public health approaches should be focused on maintaining adequate Hb values and avoiding loss of muscle function to preserve autonomy in the older adult population.

## Data availability statement

The datasets presented in this study can be found in online repositories. The names of the repository/repositories and accession number(s) can be found at: https://www.who.int/data/data-collection-tools/study-on-global-ageing-and-adult-health/sage-waves.

## Ethics statement

The studies involving humans were approved by Research and ethics committees of the National Institute of Public Health, Cuernavaca, Mexico (CI/2020/550). The studies were conducted in accordance with the local legislation and institutional requirements. The participants provided their written informed consent to participate in this study. Written informed consent was obtained from the individual(s) for the publication of any potentially identifiable images or data included in this article.

## Author contributions

VC-G: Investigation, Methodology, Writing – original draft, Writing – review & editing. AS-R: Investigation, Methodology, Writing – original draft, Writing – review & editing, Conceptualization, Data curation, Funding acquisition, Project administration, Software. BM-E: Conceptualization, Funding acquisition, Investigation, Methodology, Project administration, Writing – review & editing.
